# Pulse-wave velocity assessments derived from a simple photoplethysmography device: Agreement with a referent device

**DOI:** 10.3389/fcvm.2023.1108219

**Published:** 2023-02-07

**Authors:** Gabriel Zieff, Keeron Stone, Craig Paterson, Simon Fryer, Jake Diana, Jade Blackwell, Michelle L. Meyer, Lee Stoner

**Affiliations:** ^1^Department of Exercise and Sport Science, University of North Carolina at Chapel Hill, Chapel Hill, NC, United States; ^2^School of Sport and Exercise, University of Gloucestershire, Gloucester, United Kingdom; ^3^Department of Physiology, The University of Arizona, Tucson, AZ, United States; ^4^Department of Emergency Medicine, University of North Carolina at Chapel Hill, Chapel Hill, NC, United States

**Keywords:** arterial stiffness, oscillometry, repeatability, tilt-testing, validity, photoplethysmography

## Abstract

**Objective:**

Pulse-wave velocity (PWV), a common measure of arterial stiffness, can be measured continuously and across multiple body sites using photoplethysmography (PPG). The objective was to determine whether a simple photoplethysmography PPG PWV method agrees with a referent device.

**Approach:**

Photoplethysmography heart-finger PWV (hfPWV) and heart-toe PWV (htPWV) were compared to oscillometric carotid-wrist PWV (cwPWV) and carotid-ankle PWV (caPWV) referent measurements, respectively. In 30 adults (24.6 ± 4.8 years, body mass index 25.2 ± 5.9 kg/m^2^, 18 female), three measurements were made: two supine baseline measurements (Base 1, Base 2) and one measurement (Tilt) 5 min after a modified head-up tilt test (mHUTT). Overall agreement and repeated measures agreement (change in PPG PWV from Base to Tilt vs. change in referent PWV from Base to Tilt) were calculated using linear mixed models. Agreement estimates were expressed as intra-class correlation coefficients (ICC).

**Main results:**

For hfPWV there was strong overall agreement (ICC: 0.77, 95%CI: 0.67–0.85), but negligible and non-significant repeated measures agreement (ICC: 0.10, 95%CI: −0.18 to 0.36). For htPWV, there was moderate overall agreement (ICC:0.50, 95%CI: 0.31–0.65) and strong repeated measures agreement (ICC: 0.81, 95%CI: 0.69–0.89).

**Significance:**

Photoplethysmography can continuously measure PWV at multiple arterial segments with moderate-strong overall agreement. While further work with upper-limb PPG PWV is needed, PPG can adequately capture acute changes in lower-limb PWV.

## Introduction

Arterial stiffness, a composite measure of arterial structure and functions, is commonly assessed using pulse-wave velocity (PWV) (1–4). PWV is the velocity at which the forward-traveling pulse-wave moves between proximal and distal arterial sites (4). Typically, the pulse waveform is simultaneously or sequentially measured at two sites of interest using tonometry (pressure transducer) or oscillometry (blood pressure cuff) (5). One drawback of these methods is that they typically only permit data collection at discrete time points. Thus, they are not well-suited to acute physiological studies in which PWV is ideally assessed continuously over a defined period (e.g., to track response to an orthostatic challenge). A major advantage of continuous compared to discrete PWV measurements is the higher temporal resolution, increasing the likelihood that the “true” vascular response will be captured. A viable approach which would permit continuous PWV measurement, if found to be repeatable and in agreement with an established referent device, is photoplethysmography (PPG). PPG reflects oscillations in the microcirculation, which is governed by both local and central (autonomic) systems (6).

Several studies have compared PPG-based PWV assessments to traditional PWV techniques with mixed findings (i.e., *r* = 0.34–0.95) (7–11). The fact that these studies failed to consistently demonstrate strong relationships between PPG and criterion PWV techniques is likely partially attributed to the fact that these studies compared different arterial segments, and thus are not directly comparable. To extend the utility of PPG-based PWV measurements in biomedical research, it is necessary to determine if PPG assessments agree with a referent method at an analogous arterial segment. Additionally, it would be helpful to determine whether PPG-based PWV can track acute changes in arterial stiffness. Static agreement, which we refer to as “overall” agreement, does not determine whether a device is capable of tracking change in a desired construct. Alternatively, more meaningful information can be determined by ascertaining whether a change in one measure agrees with change in the other measure, which we refer to as “repeated measures” agreement.

The purpose of this study was to compare PPG-based heart-finger PWV (hfPWV) and heart-toe PWV (htPWV) with referents carotid-wrist PWV (cwPWV) and carotid-ankle PWV (caPWV), respectively. The objectives were to determine the: (i) overall agreement (independent of condition), and the (ii) repeated measures agreement. Repeated measures agreement was tested by determining whether the change in PWV from supine to modified head-up tilt test (mHUTT) postures was comparable across device. We selected mHUTT because is induces rapid, robust autonomic responses which are known to influence PWV (12–15). Collectively, the findings will provide important information about the potential utility of a novel, simple strategy to continuously assess PWV.

## Materials and methods

This study is reported in accordance with Consolidated Standards of Reporting Trials guidelines (16).

### Ethical approval

Ethical approval was obtained from the University of North Carolina at Chapel Hill Office of Human Research Ethics (19-0828) and participants provided written informed consent.

### Participants

Thirty young healthy women and men were recruited from a large public university. A young, (18–40 years) healthy sample was recruited to mitigate the risk of age- or disease-related influences on the primary outcomes. Participants were excluded if they reported any known cardiometabolic disorders (e.g., type II diabetes, coronary artery disease), were taking medications known to affect cardiovascular function or reported smoking. This study was not designed to examine sex differences; thus, menstrual cycle was not controlled for in females.

### Experimental design

A single-visit, two-condition design was employed, in which PPG-based hfPWV and htPWV were compared to referent measures (cwPWV and caPWV, respectively). Measurements were made while supine and following mHUTT. cfPWV was included since it is the criterion PWV measure used most commonly for clinical and prognostic purposes (3, 17) and demonstrates excellent reliability (18, 19). Peripheral blood pressure (BP) were captured to confirm that mHUTT induced the desired autonomic perturbation.

All measurements were collected in a quiet, environmentally controlled room (average: 22°C, 50% humidity, 748 mmHg). Participants arrived between 600 and 1,000 having fasted for 12 h and having consumed only water that morning. Additionally, participants were asked to avoid strenuous physical activity and alcohol for 24 h prior to experimentation. Following anthropometric assessments, the test device PPG sensors (IR Plethysmograph, ADInstruments) and referent Vicorder cuffs (SMT Medical, Wuerzburg, Germany) were positioned on the arterial sites of interest and electrocardiogram (ECG) with electrodes placed on the chest. Arterial pathlengths were measured using a custom device to bypass body contours while participants lay in a supine position (20).

Measurements were collected continuously for PPG. For the Vicorder, semi-automated PWV measurements were made in triplicate. Following a 10-min rest period in which the participant was at a slightly elevated (30°) position to prevent venous backflow, two sets of measures (Base 1, Base 2) were taken 10 min apart. Since PPG and Vicorder measurements occurred simultaneously, this 30° elevated position was used for Base 1 and Base 2 assessments for both PPG and Vicorder devices. For the Vicorder, cwPWV and caPWV were measured first (e.g., before cfPWV and BP) as accurate assessment of these measures were imperative since they served as the referent for PPG hfPWV and htPWV comparisons, respectively.

### Modified head-up tilt test

Following the slightly-elevated (30°) Base 1 and 2 Vicorder measurements, the participant was lowered to a completely supine position (0°) for 2 min to allow for a return to autonomic “baseline” prior to mHUTT. Subsequently, the mHUTT was administered in which the participant was quickly raised to a seated (78° angle) position. Following 5 min of rest in the 78° seated mHUTT position, all measurements were repeated (Tilt). In accordance with ours and colleagues’ previous work (12, 13, 15, 21, 22), this 5-min interim period was used to allow for a new physiological “set-point” or “steady-state” to be reached.

### Experimental measures

The full PPG and Vicorder setup is described below and shown in Supplementary Figure 1.

#### Referent device: Vicorder

**FIGURE 1:**
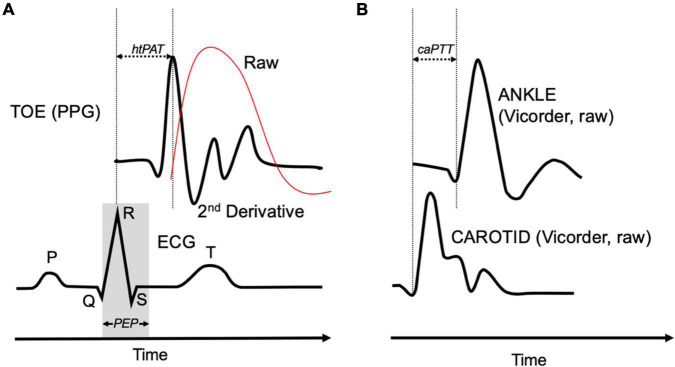
**(A)** Heart-toe pulse arrival time (htPAT) used for heart-toe pulse-wave velocity (htPWV) calculation (red line = raw PPG signal) and **(B)** carotid-ankle pulse transit time (caPTT) used for carotid-ankle pulse-wave velocity (caPWV) calculation. Alignment of raw foot and 2nd derivative peak. ECG, electrocardiogram; PEP, pre-ejection period; PPG, photoplethysmography.

The Vicorder was used to measure cwPWV, caPWV, cfPWV, and BP (23, 24). BP cuffs were placed around the arm, wrist, thigh, and ankle. The neck cuff was placed over the carotid artery with the bladder positioned where the pulse was strongest. The arm cuff was placed over the brachial artery. The wrist cuff was placed over the radial artery immediately proximal to the pisiform bone. The thigh cuff was placed over the femoral artery as high up on the leg as possible. The ankle cuff was placed over the posterior tibial artery with the distal portion of the cuff placed immediately above the malleolus. PWV (m/s) was calculated by dividing arterial path length (D) by the pulse transit time (TT) between the mid-point of the proximal (carotid) and distal cuffs. D was calculated by subtracting the distance between the suprasternal notch (SSN) and the mid-point of the carotid cuff from the distance between the proximal and distal segment. To measure TT, the two cuffs were simultaneously inflated to a low (∼50 mmHg) pressure, and a proprietary algorithm calculated the TT between the foot of the proximal and distal pressure waveforms (Figure 1). This algorithm has built-in adjustments that account for the time-delays associated with the measurement of pressure changes and thus does not include the pre-ejection period. Three measurements were taken at each time-point (Base 1, Base 2, and Tilt) and the average of the closest two measures were analyzed.

#### Test device: Photoplethysmography

Photoplethysmography and ECG were used to measure htPWV and hfPWV. The PPG device captures pulse waveforms by emitting an infrared light at a 940 mm wavelength. Analog signals were transmitted and digitized using the data acquisition system (Powerlab, AD Instruments). The PPG sensors were placed on the (1) index fingertip and (2) tip of the big toe. A standard three-lead ECG was placed on the chest. ECG and PPG signals were sampled at 1,000 Hz, providing a temporal resolution of 1 ms. For the D measurements, the SSN and mid-point of the PPG probes were used as the proximal and distal sites, respectively. For hfPWV, D was measured directly from the SSN to the mid-point of the finger cuff. For htPWV, D was measured as the sum of the distances between the SSN and umbilicus, the umbilicus and malleolus, and the malleolus and mid-point of the PPG toe sensor. The time component for the PPG PWV equation, PAT, originates from the ECG signal and includes the pre-ejection period. PAT, which is the duration from the R wave to the 2nd derivative peak of the PPG, is depicted in Figure 1. The R wave was used as the starting point of PAT as it closely aligns with ventricular ejection (25). Moreover, the R wave is prominent and typically provides an adequate signal even amidst participant movement or signal artifact. The foot of the PPG waveform was identified using the 2nd derivative peak as it closely aligns with the ECG R wave peak as depicted in Figure 1 (schematic) and Supplementary Figure 2 (actual data trace from Labchart software). Powerlab and LabChart data processing details are also described in the Supplementary Table 1.

### Randomization

There was potential for the sub-systolic pressure inflations from the Vicorder to interfere with the PPG signal if the devices were placed on the same side of the body. For example, because the Vicorder cuffs were placed proximal relative to the PPG cuff for each measurement site (e.g., for upper limb, Vicorder cuff was placed at wrist, whereas PPG cuff was placed distal to this location at the finger tip), if both devices were placed on the same limb, Vicorder cuff inflations would likely have altered the speed and morphology of the pressure wave captured by the downstream PPG cuff. A potential solution that would allow for both devices to be placed on the same side of the body would have been to take consecutive measurements with each device. However, given the importance of comparing device measurements during the same cardiac cycle and resulting pressure wave, it was critical for Vicorder and PPG measurements to occur simultaneously. Thus, Vicorder cuffs were placed on one side of the body (all on left OR all on right), and the PPG cuffs were placed on the opposite side of the body. The order of placement was randomized using an online randomization tool.^1^ The order of conditions (Base 1, Base 2, and Tilt) were not randomized because the autonomic perturbation induced by the mHUTT would likely confound a true resting baseline assessment if it preceded the Base measures.

### Sample size

Sample size estimates were conducted using G*Power 3. A sample size of 53 [52 degrees of freedom (df)] can detect a 1-tailed correlation of 0.33 with 80% power and a 5% chance of type 1 error. We elected to use a mixed model for calculating agreement as this model enables repeated measures to be nested within each participant. Using this approach, the degrees of freedom is equal to N(k-1)-1, where N is the total number of participants and k is the number of repeated measurements per participant. This approach offers the advantage of minimizing between-subject variance and limiting the need for a greater sample size whilst maintain statistical power. Consequently, a sample size of 27 with 3 repeated measures equated to the necessary degrees of freedom and power [i.e., 27(3-1)–1 = 53]. We recruited 30 participants with the assumption that 10% of participants (*n* = 3) would either not report for testing, or the collected data would be of insufficient quality.

### Statistical analyses

Statistical analyses were performed using RKWard (Version 0.7.1). The significance level was set *a priori* at α = 0.05. Raw data are presented as mean [standard deviation (SD)] and mixed model data are presented as intraclass correlation coefficients [(ICC); 95% confidence interval (95%CI)]. The corresponding author (GZ) had full access to the data in the study and was responsible for the integrity of the data set and the data analysis.

Paired *t*-tests were used to compare baseline (average of Base 1 and 2) and Tilt responses. Effect sizes (ES) were calculated as Cohen’s *d* where <0.2 was defined as trivial, 0.2–0.3 as small, 0.4–0.8 as moderate, and >0.8 as large (26). For repeatability, ICC estimates and their 95% CIs were determined using mean-centered, absolute-agreement, 2-way mixed-effects models. A mixed model was used as it is unaffected by sample size (27). Although there is no universal standard for classifying the magnitude of ICC, for criterion-related assessments: values less than 0.50 are indicative of poor repeatability, values between 0.50 and 0.75 indicate moderate repeatability, values between 0.75 and 0.90 indicate good repeatability, and values greater than 0.90 indicate excellent repeatability (28). A standard error of measurement (SEM) was also calculated according to the formula: SD × √(1-ICC) and a minimal detectable change (MDC) was calculated according to the formula: 1.96 × SEM × √2 (29). MDC% was calculated as (MDC/mean) × 100, and is the MDC relative to the mean measurement value.

Agreement between PPG and referent measures were determined using a series of linear mixed-effects models. The mixed-model approach maximizes statistical power while accounting for the correlated error variances and the condition (Base, Tilt) structure. To calculate the overall measurement agreement, cwPWV and caPWV were regressed against hfPWV and htPWV, respectively, and nested within subject. Subject and Condition intercepts were specified as random effects and used to estimate the between-subject (σ^2^_*s*_), repeated measures (σ^2^_*c*_) and residual (σ^2^_*r*_) variance. Subsequently, the overall (independent of condition) ICC was calculated as σ^2^_*s*_ / (σ^2^_*s*__+_ σ^2^_*r*_). To calculate repeated measures agreement (strength of association for change in one device versus change in other device; e.g., between Base and Tilt conditions), we used the repeated measures correlation (rmcorr) package for R (30). The rmcorr statistical technique determines the overall within-individual relationship among paired measures assessed on two or more occasions (30). Model assumptions were tested using Q-Q plots to diagnose normal distribution, and by plotting residual against fitted values to inspect bias. Although there is no universal criterion for adjudicating the strength of agreement, estimates were defined as negligible (<0.2), weak (0.2), moderate (0.4), strong (0.7), or very strong (0.9) (31).

We used the approach outlined by Parker et al. (32) to calculate the absolute difference between the two measures and assess the uniformity of error. In terms of the model used to assess error parameters, PWV (i.e., all PPG and referent measurements) was specified as the dependent variable nested within subject and condition, and device was set as a fixed factor. The following random effects were specified to calculate the variance (σ^2^) components: subject (σ^2^_γ_), condition (σ^2^_α_), subject-condition (σ^2^_αβ_), subject-measure (σ^2^_αγ_), measure-condition (σ^2^_βγ_), and residual (σ^2^*_ε_*). Subsequently, the absolute difference between measure was estimated by calculating the square root of the mean squared difference ($\sqrt{\text{MSD}}$). The coverage probability (CP) determined the estimated proportion of values which fell within the clinically acceptable difference (CAD). Mixed effects limits of agreement plots were generated to inspect and test the uniformity of error. Last, due to: (i) slightly differing path lengths (D) between PPG and referent devices and (ii) the inclusion of the pre-ejection period in the PPG-based time component (PAT) but not the referent-based time component (TT), we did not expect absolute agreement between devices. Therefore, the above steps were repeated after correcting hfPWV and htPWV using the mixed effects regression model (32–34).

## Results

### Participants

Data was collected on 25 of the 30 participants (24.8 years [SD 5.0], 15 female, 25.2 kg/m^2^ [SD 5.9]). Of the five remaining participants, data was entirely omitted for three individuals, while two contributed partial data. Excluded data was a result of poor Vicorder or ECG data.

### Within-day reliability (repeatability)

Repeatability data are reported in Table 1. All measures exceed the criterion for acceptable repeatability.

**TABLE 1:** Repeatability of baseline pulse-wave velocity measurements [Data points = 54 (27 subjects × 2 time points)].

	ICC (95%CI)	SEM m/s (95%CI)	MDC m/s (95%CI)	MDC % (95% CI)
cwPWV	0.99 (0.97–0.99)	0.05 (0.08–0.03)	0.14 (0.10–0.21)	2.66 (1.79–3.95)
caPWV	0.98 (0.95–0.99)	0.16 (0.23–0.11)	0.44 (0.29–0.64)	6.51 (4.38–9.65)
cfPWV	0.97 (0.93–0.99)	0.09 (0.14–0.06)	0.26 (0.18–0.39)	3.62 (2.44–5.34)
hfPWV	0.97 (0.93–0.99)	0.06 (0.09–0.04)	0.16 (0.11–0.24)	5.04 (3.39–7.45)
htPWV	0.95 (0.90–0.98)	0.09 (0.14–0.06)	0.26 (0.18–0.38)	5.94 (4.01–8.75)

caPWV, carotid-ankle pulse-wave velocity (Vicorder); cfPWV, carotid-femoral pulse-wave velocity (Vicorder); cwPWV, carotid-wrist pulse-wave velocity (Vicorder), CI, confidence interval; ICC, intra-class correlation coefficient; hfPWV, heart-finger pulse-wave velocity (photoplethysmographpy); htPWV, heart-toe pulse-wave velocity (photoplethysmography); MDC, minimal detectable change; m/s, meters per second; SEM, standard error of measurement.

### Modified-tilt response

BP and PWV responses to the mHUTT are reported in Table 2. There was a large effect size (>0.8) increase in mean arterial pressure (MAP) (7.9 mmHg, 95%CI: 6.1–9.5) and a large increase in cfPWV (1.9 m/s, 95%CI: 1.5–2.2). There were also large increases in caPWV (1.5 m/s, 95%CI: 1.4–1.8) and htPWV (0.4 m/s, 95%CI: 0.32–0.52). However, while there was a non-significant increase in cwPWV (0.1 m/s, 95%CI: −0.12 to 0.33) there was a large decrease in hfPWV (−0.1 m/s, 95%CI −0.08 to −0.18).

**TABLE 2:** Pulse-wave velocity response to the modified head-up tilt-table test responses [Data points = 81 (27 subjects × 3 time points)].

		MAP	cfPWV	cwPWV	caPWV	hfPPW	htPWV
		**mm Hg**	**m/s**	**m/s**	**m/s**	**m/s**	**m/s**
Mean	Base	76.9	5.6	6.6	7.3	3.3	4.4
	Tilt	84.8	7.5	6.7	8.8	3.2	4.8
SD	Base	6.1	0.8	0.9	0.5	0.4	0.5
	Tilt	8.1	1.2	0.9	0.5	0.4	0.5
	*P*	<0.001	<0.001	0.386	<0.001	<0.001	<0.001
	ES	1.88	2.01	0.20	3.08	0.99	1.67

ES, effect size; MAP, mean arterial pressure; mmHg, millimeters of mercury; m/s, meters per second; SD, standard deviation.

### Measurement agreement

**FIGURE 2:**
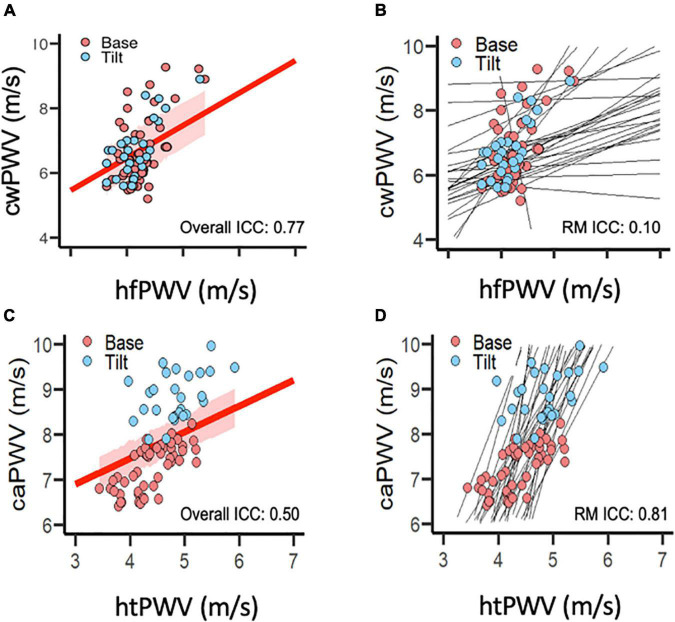
Agreement between **(A,B)** carotid-wrist pulse-wave velocity (referent) and heart-finger pulse-wave velocity (photoplethysmography), and between **(C,D)** carotid-ankle pulse-wave velocity (referent) and heart-toe pulse-wave velocity (photoplethysmography). **(A,C)** Intra-class correlation for overall agreement (independent of condition) between devices. Red line represents overall correlation between devices; shaded red area represents 95% confidence interval. **(B,D)** Intra-class correlation for repeated measures agreement (between-condition change from Base to Tilt) between devices. (photoplethysmography). Black lines represent within-subject, repeated measures correlations. [Data points per plot = 81 (27 subjects × 3 time points)]. caPWV, carotid-ankle pulse-wave velocity; cwPWV, carotid-wrist pulse-wave velocity; hfPWV, heart-finger pulse-wave velocity; htPWV, heart-toe pulse-wave velocity; ICC, intra-class correlation coefficient; m/s, meters per second; RM, repeated measures (agreement).

For the mixed model, the Q-Q plots indicated normal distribution, and the plot for predicted values against the residuals indicated no bias. The agreement parameters are reported in Table 3. Figure 2 presents the overall and repeated measures comparisons between hfPWV and cwPWV, and between htPWV and caPWV, respectively. For hfPWV vs. cwPWV, there was strong overall agreement (ICC: 0.77, 95%CI: 0.67–0.85), but low repeated measures agreement (ICC: 0.10, 95%CI: −0.18 to 0.36). For htPWV vs. caPWV, there was moderate overall agreement (ICC: 0.50, 95%CI: 0.31–0.65), and strong repeated measures agreement (ICC: 0.81, 95%CI: 0.69–0.89).

**TABLE 3:** Measurement agreement parameters including uncorrected and corrected results (Total data points = 81).

	Mean	(SD)	Mean	(SD)	ICC (95% CI)		$\sqrt{\text{M}\,S\,D}$	CP
	**cwPWV (m/s)**		**hfPWV (m/s)**				**m/s**	**%**
**Uncorrected**								
Overall	6.7	(1.0)	3.2	(0.3)	0.77	(0.67 to 0.85)	3.62	22
Repeated measures	0.1	(0.6)	−0.1	(0.1)	0.10	(−0.18 to 0.36)		
**Corrected**								
Overall	6.7	(1.0)	6.7	(0.9)	0.77	(0.67 to 0.85)	1.31	56
Repeated measures	0.1	(0.6)	−0.1	(0.1)	0.09	(−0.18 to 0.36)		
**Uncorrected**								
Overall	7.8	(0.9)	4.5	(0.5)	0.50	(0.31 to 0.65)	3.33	24
Repeated measures	1.6	(0.5)	0.4	(0.3)	0.81	(0.69 to 0.89)		
Corrected								
Overall	7.8	(0.9)	7.8	(0.6)	0.50	(0.31 to 0.65)	0.59	91
Repeated measures	1.6	(0.5)	0.6	(0.3)	0.81	(0.69 to 0.89)		

Due to: (i) slightly differing path lengths between photoplethysmography and referent devices and (ii) the inclusion of the pre-ejection period in the photoplethysmography-based time component but not the referent-based time component, we did not expect absolute agreement between devices. Therefore, to permit more direct comparison between devices, the “corrected” data refers to results after correcting hfPWV and htPWV using the mixed effects regression model. caPWV, carotid-ankle pulse-wave velocity; CI, confidence interval; CP, coverage probability; cwPWV, carotid-wrist pulse-wave velocity; hfPWV, heart-finger pulse-wave velocity; htPWV, heart-toe pulse-wave velocity; ICC, intra-class correlation coefficient; SD, standard deviation; MSD, square root of the mean squared difference.

### Measurement error

**FIGURE 3:**
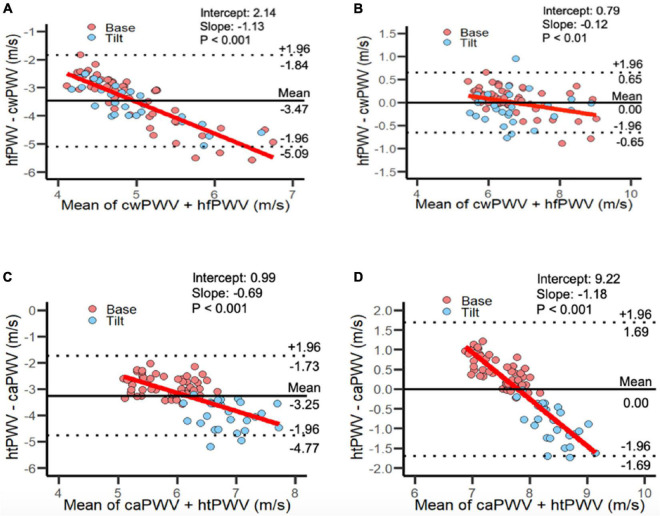
Limits of Agreement plots for panel **(A)** uncorrected and **(B)** corrected heart-finger pulse-wave velocity vs. carotid-wrist pulse-wave velocity; combined Base and Tilt. [81 data points per plot: (27 subjects × 3 time points)]. Limits of Agreement plots for panel **(C)** uncorrected and **(D)** corrected heart-toe pulse-wave velocity and carotid-ankle pulse-wave velocity; combined Base and Tilt: [81 data points per plot: (27 subjects × 3 time points)]. caPWV, carotid-ankle pulse-wave velocity; cwPWV, carotid-wrist pulse-wave velocity; hfPWV, heart-finger pulse-wave velocity; htPWV, heart-toe pulse-wave velocity.

To permit more direct comparison of hfPWV to cwPWV, the hfPWV values were corrected using mixed model regression (Table 3). For the comparison between upper limb measurements (hfPWV vs. cwPWV), the corrected variance component estimates were: σ^2^_α_ (condition) = 0.00; σ^2^_γ_ (subject) = 0.81; σ^2^_αβ_ (subject-condition) = 0.02; σ^2^_αγ_ (subject-measure) = 0.00; σ^2^_βγ_ = (measure-condition) = 0.00, and σ^2^*_ε_* (residual) = 0.05. As such, the subject variable explained a considerable source of the overall variation, while the negligible subject-device component indicates no evidence of a difference in the measure effect across subjects. The corrected $\sqrt{\text{MSD}}$ was 1.31 m/s, which is greater than the *a priori* CAD of 1 m/s. The CP indicates that 56% of the difference values are below 1 m/s. Inspection of the LoA (Figure 3) indicates proportional error, which is confirmed by the significant and negative slope (β = −0.12, *P* < 0.01).

For the comparison between lower limb measurements (htPWV vs. caPWV), the corrected variance component estimates were: σ^2^_α_ (condition) = 0.14; σ^2^_γ_ (subject) = 0.08; σ^2^_αβ_ (subject-condition) = 0.00; σ^2^_αγ_ (subject-measure) = 0.05; σ^2^_βγ_ (measure-condition) = 0.28, and σ^2^*_ε_* (residual) = 0.05. As such, condition and the measure-condition interaction explained a considerable source of the overall variation. The subject-measure interaction was negligible, indicating no evidence of a difference in the measure effect across subjects. The corrected $\sqrt{\text{MSD}}$ was 0.59 m/s which is less than the *a priori* established CAD of 1 m/s. The CP indicates that 91% of the difference values are below 1 m/s. However, inspection of the LoA (Figure 3) indicates proportional error, which is confirmed by the significant and negative slope (β = −1.18, *P* < 0.001)

### Ancillary analysis

To ensure full transparency, ancillary analysis in the Supplementary Reports associations between devices where PPG PWV was calculated using raw and first derivative signals (Supplementary Table 2).

## Discussion

The objective of the current study was to assess the agreement between a reference oscillometric device and a simple, non-invasive PPG device for measurement of PWV. For the upper-limb segments, there was strong overall agreement and negligible repeated measures agreement between PPG hfPWV and the referent cwPWV. The poor repeated measures agreement between hfPWV and cwPWV was likely attributed to the lack of repeated measures (Base vs. Tilt) change in cwPWV. For the lower-limb segments, there was moderate overall agreement and strong repeated measures agreement between PPG htPWV and the referent caPWV.

### Limitations and strengths

Several limitations should be acknowledged. First, a young, healthy sample was recruited to mitigate the risk of age or disease. Future studies with older and clinical populations are now required to better generalize the findings. Second, we unintentionally tested an all-white sample. As melanin concentration of the skin impacts PPG signal quality (35), further work is needed to assess the acceptability of PPG PWV in non-white individuals. There were also several strengths of the current study. First, we used an established oscillometric method as the referent to test the agreement of the PPG method. Second, we filled a gap in the literature by investigating agreement with an accepted measure, and additionally assessed repeated measures agreement.

### Comparison to literature

In terms of agreement with the referent in the current study, hfPWV exhibited strong overall agreement with the referent cwPWV. In contrast, PPG htPWV had only moderate overall agreement with the referent caPWV. Tsai et al. (7) and Cho et al. (8) conducted the only prior studies comparing PPG against criterions. Tsai et al. (7) reported a moderate correlation (*r* = 0.68, *p* < 0.01) between PPG finger-toe PWV and tonometric cfPWV as well as higher PWV values (from both devices) in subjects with hypertension and dyslipidemia, while Cho et al. (8) showed a strong correlation in females (*r* = 0.79, *p* < 0.01) but not males (*r* = 0.34, *p* = 0.34), between PPG hfPWV and oscillometric brachial-ankle PWV. The reason for the sex-differences observed by Cho et al. (8) are unclear, and while the current study was not powered to be able to decipher sex-differences, future work exploring potential sex discrepancies in PPG PWV is warranted. It may be the case that greater progression of CVD risk among males contributed to the observed differences in the association between PPG and traditional PWV. Sex differences aside, intrinsic differences in path length used in PWV in each study, including ours, may partially explain these mixed results. The literature would benefit from future studies that compare PPG and traditional PWV methods with identical pathlengths.

We also extend the literature by assessing repeated measures agreement (e.g., change from Base to Tilt conditions). Whereas htPWV demonstrated strong repeated measures agreement with the referent caPWV, there was negligible repeated measures agreement between hfPWV and the referent cwPWV. This is not completely unexpected since the shorter path length of hfPWV is inherently susceptible to greater relative error than that of htPWV, therefor influencing measurement agreement to a greater extent (34). While a recent study by Ouyang did not compare PPG measures against a referent or use an acute laboratory perturbation, they did observe expected, incremental increases in PPG heart-toe PWV with both increasing age and elevated disease status (36). Interestingly, however, they did not observe this trend, and saw greater within-subject variability, with PPG heart-finger PWV compared to heart-toe PWV (36). Their findings were interpreted to be a function of elastic artery stiffness being more reflective of CVD risk compared to muscular artery stiffness (36). This explanation is in line with the understanding of the arterial stiffness gradient and its association with CVD risk (37), including knowledge obtained from a recent epidemiological investigation by our group (38). Further, Ouyang et al.’s (36) findings parallel our own results of strong repeated measures agreement in the lower, but not the upper-limb arterial segments.

While the differences in elastic vs. muscular arteries may be one explanation for the lack of repeated measures agreement between hfPWV and cwPWV, another reason may simply be the lack of repeated measures (Base vs. Tilt) change in the latter. The lack of change from Base to Tilt in cwPWV would seem to imply that we failed to induce an increase in arterial stiffness. However, we likely did induce an increase in central arterial stiffness, given the increase in MAP (ES: 1.88) and cfPWV (ES: 2.01). Previous studies have demonstrated changes in PWV following a positional perturbation (39, 40), suggesting that these perturbations can affect arterial stiffness. However, further work is needed to better understand the lack of repeated measures agreement between hfPWV and cwPWV observed in the current study. It may be the case that the upper limbs may simply not need to respond to a whole-body orthostatic challenge in order to maintain adequate perfusion of vital organs (e.g., heart, brain).

### Implications

The present research is one of few studies that have (i) assessed the agreement between PWV derived from PPG and a referent device and (ii) provided sufficient detail to permit replication (i.e., Supplementary Table 1). Since baseline agreement only provides limited pathophysiological insight, we leveraged the high temporal resolution and continuous nature of PPG by measuring repeated measures agreement in response to mHUTT. For example, assessing acute, continuous changes in vascular function in response to a psychological stressor or a bout of physical exercise can sensitively reflect cardiovascular reactivity, which confers additional prognostic information beyond baseline assessments (41–46). The high temporal resolution and continuous nature of PPG further allows for structural and functional comparisons between distinct arterial segments simultaneously. This unique capacity may help to provide insight into how certain arterial segments may adapt (i.e., in response to chronic aerobic exercise training) or deteriorate (i.e., in response to aging, smoking, physical inactivity, or sedentary behavior) distinctly. While the continuous nature of PPG does provide important physiological insight, it should equally be emphasized that no clinical relevance can be gleaned from PPG-derived PWV at present. A 1 m/s change in PWV is generally considered clinically significant (47). However, the interpretation of a 1 m/s change as clinically significant is generally reserved for situations in which this change is (i) a result of chronic age- or disease-related processes (rather than acute reactivity to a perturbation) and (ii) assessed via tonometry- or oscillometry-derived PWV.

### Future directions

Future studies are needed to assess the utility of PPG PWV in other demographics and to determine whether PPG is a suitable technique to assess more chronic vascular changes (e.g., due to disease or exercise training). While our findings are noteworthy, it would also be meaningful to test repeated-measures agreement against established path-lengths such as with cfPWV, which is a true criterion and has important prognostic utility. Nevertheless, our PPG-derived PWV measurements did capture major segments of the central arterial system. Further, stiffness indices which include peripheral segments, such as the PPG PWV measures assessed in the current study, may be particularly relevant to elucidating important mechanistic information related to lifestyle behaviors linked to cardiovascular disease risk [e.g., our group has shown that lower-limb PWV increases with exposure to sedentary behavior (48), an independent risk factor for CVDs (49)].

## Conclusion

The objective of the current study was to assess the agreement between a traditional oscillometric PWV device and a novel PWV method using a simple, non-invasive PPG device. Our findings show that PPG can continuously measure PWV at multiple arterial segments with acceptable repeatability. However, while PPG may be suitable for simple upper-limb assessments of PWV, its utility may be limited in scenarios in which there is a need to assess the upper-limb vasculature’s reactivity to acute laboratory perturbations. In contrast, PPG can adequately capture acute changes in lower-limb PWV in response to laboratory perturbations. These findings suggest that PPG-based PWV may be a viable tool for continuous PWV measurement, but will benefit from future methodological adjustments. Future studies are warranted to further compare PPG PWV against criterion devices as and explore the prognostic capacity of PPG PWV measurements.

## Data availability statement

The raw data supporting the conclusions of this article will be made available by the authors, without undue reservation.

## Ethics statement

The studies involving human participants were reviewed and approved by University of North Carolina at Chapel Hill Institutional Review Board. The patients/participants provided their written informed consent to participate in this study.

## Author contributions

GZ and LS conceived the research question and study design. GZ, KS, CP, SF, JD, JB, and LS were responsible for data collection. GZ, KS, CP, SF, and LS analyzed and interpreted the data. GZ led the writing of the manuscript with critical editing and proofing from the remaining authors. All authors contributed to the article and approved the submitted version.

## Footnotes

1
www.randomizer.org
