# Supplementary material for: Pulse-wave velocity assessments derived from a simple photoplethysmography device: Agreement with a referent device
Source: Front Cardiovasc Med. 2023 Feb 7;10:1108219. doi: 10.3389/fcvm.2023.1108219 (PMC9941627; doi:10.3389/fcvm.2023.1108219)
Supplement: Supplementary file 1 [file Table_1.DOCX]

**SUPPLEMENT**

**TABLE OF CONTENTS**

**Table S1**: Powerlab and Labchart channel settings………………………………………………….2

**Table S2**: Device agreement using raw and 1st derivative peaks for pulse arrival time calculation ………………………………………………………………………………………………...3

**Figure S1**: Full setup of photoplethysmography and referent Vicorder devices. *…………………4*

**Figure S2**: LabChart data example of overlaid raw and second derivative photoplethysmography signals………………………………………………………………………….5

**Custom LabChart macro code:** For determination of pulse arrival time and subsequent photoplethysmography pulse-wave velocity calculation……………………………………………...6

### **Data processing**…………………………………………………………………………………………8

**Measurement considerations**………………………………………………………………………..10

# TABLE S1. PowerLab and LabChart channel settings

| Channel Type | Device Input | Sample Rate | Range | Calculation/Filters/Detection Settings Pre-set | Source Channel | Other |
| --- | --- | --- | --- | --- | --- | --- |
| ECG | PowerLab | 1k/s | 100 mV | Digital filter (low-pass, 50-Hz cut-off), no calculation | N/A | N/A |
| PPG Raw | PowerLab (no calculation) | 1k/s | 10 V | No calculation | N/A | N/A |
| PPG Derivative channels (e.g. 1st, 2^nd^) | Calculation | N/A | N/A | Auto-scale, derivative calculation | PPG raw | Adjust derivative order, window width, amplitude axis, and decimal places as needed |
| PPG Cyclical | Calculation | N/A | N/A | Preset: Cardiovascular-Arterial Pressure; check “event markers” | PPG derivative | Adjust minimum peak height and standard deviation (start SD: 2) as needed |

PowerLab and Labchart channel settings used for photoplethysmography and electrocardiogram data collection and reduction. **Abbreviations:** *ECG, electrocardiogram; Hz, hertz; k/s, 1000 per second; mV, millivolts; N/A, not applicable; PPG, photoplethysmography; SD, standard deviation.*

# TABLE S2. Device agreement using raw and 1st derivative peaks for pulse arrival time calculation.

|  | Referent | | Test |  | ICC | |  |  |
| --- | --- | --- | --- | --- | --- | --- | --- | --- |
| Agreement | Mean | (SD) | Mean | (SD) | (95% CI) | | |  |
| hfPWV vs. cwPWV - raw peak | | | | | | | | |
| Overall | 6.7 | (1.0) | 1.9 | (0.4) | 0.85 | (0.78 - 0.90) | |  |
| Repeated Measures | 0.1 | (0.6) | 0.1 | (0.0) | 0.30 | (0.09 - 0.49) | |  |
| htPWV vs. caPWV - raw peak | | | | | | | | |
| Overall | 7.8 | (0.9) | 2.9 | (0.4) | 0.66 | (0.52 - 0.77) | |  |
| Repeated Measures | 1.6 | (0.5) | 0.1 | (0.3) | 0.93 | (0.89 - 0.96) | |  |
| hfPWV vs. cwPWV - 1st derivative peak | | | | | | | | |
| Overall | 6.7 | (1.0) | 2.8 | (0.3) | 0.77 | (0.66 - 0.85 | |  |
| Repeated Measures | 0.1 | (0.6) | -0.1 | (0.2) | 0.06 | (-0.16 - 0.28) | |  |
| htPWV vs. caPWV - 1st derivative peak | | | | | | | | |
| Overall | 7.8 | (0.9) | 3.9 | (0.4) | 0.57 | (0.40 - 0.70) | |  |
| Repeated Measures | 1.6 | (0.5) | 0.3 | (0.1) | 0.91 | (0.86 - 0.94) | |  |

Associations between devices when photoplethysmography pulse-wave velocity were calculated using the peak derived from the raw signal and from the 1^st^ derivative (n=81, 27 subjects x 3 measurements). **Abbreviations:** *caPWV, carotid-ankle pulse-wave velocity;* *CI, confidence interval; cwPWV, carotid-wrist pulse-wave velocity; hfPWV, heart-finger pulse-wave velocity, htPWV; heart-toe pulse-wave velocity; ICC, intraclass correlation coefficient; SD, standard deviation.*

**FIGURE S1.** Full setup of photoplethysmography and referent Vicorder devices.

**
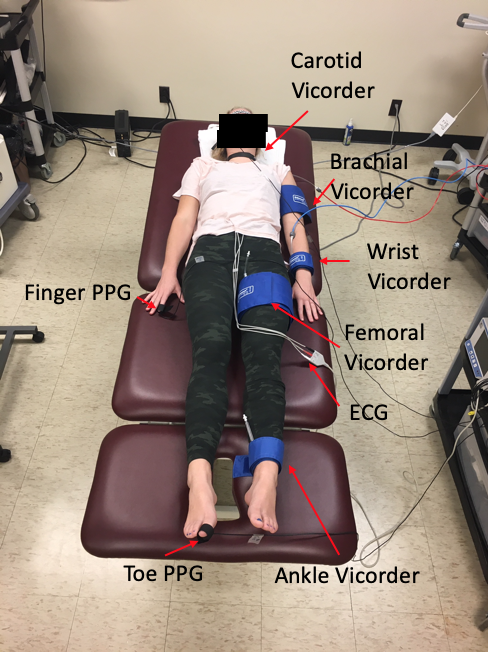
**

**Abbreviations:** *ECG, electrocardiogram; PPG, photoplethysmography.*

**Figure S2**: LabChart data example of overlaid raw and second derivative photoplethysmography signals

#
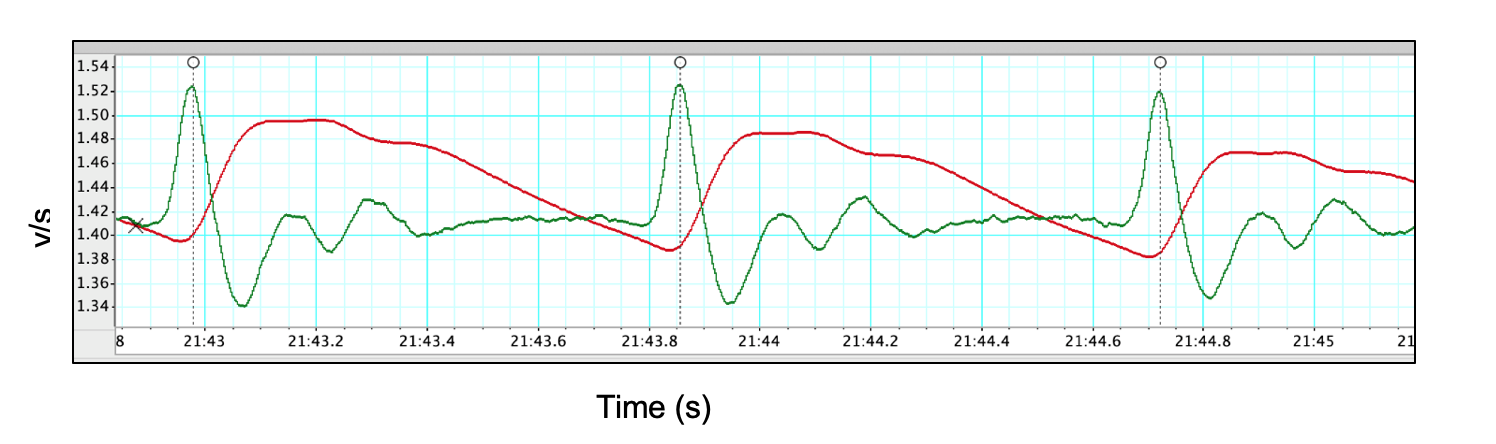


Example LabChart view of overlaid raw (red) and 2^nd^ derivative (green) photoplethysmography signals. Note alignment of raw foot and 2^nd^ derivative peak. **Abbreviations:** *S, seconds; V/s, volts per second.*

Custom LabChart macro code for calculation of photoplethysmography pulse-wave velocity

‘Call/begin macro

Sub FINGER ()

'no loop, set cursor

'auto add titles to datapad

Call Doc.DataPadAddTitles(1)

For i = 1 to 3

'Find ECG peak using Event Markers, Select single point

' Begin Find

ChannelIndex = 0

SetAction = kSetActivePoint

SelectMode = kSelectAround

SelectTime = 1

DataDisplayMode = kViewDataVisible

SelectAll = True

Direction = kSearchForward

FindType = "Search for event marker"

FindData = "JustThisChannel=1;"

Call Doc.Find (ChannelIndex, SetAction, SelectMode, SelectTime, DataDisplayMode, SelectAll, Direction, FindType, FindData)

' End Find

'Add comment peak found here

'Call Doc.Add Comment At InsertionPoint (0, "ECG")

'Find Finger 2nd Peak using Event Markers, Select to previous point

' Begin Find

ChannelIndex = 2 'channel # -1

SetAction = kSetToPreviousPoint

SelectMode = kSelectAround

SelectTime = 1

DataDisplayMode = kViewDataVisible

SelectAll = True

Direction = kSearchForward

FindType = "Search for event marker"

FindData = "JustThisChannel=1;"

Call Doc.Find (ChannelIndex, SetAction, SelectMode, SelectTime, DataDisplayMode, SelectAll, Direction, FindType, FindData)

' End Find

'Add comment peak found here

'Call Doc.AddCommentAtEnd (2, "Finger channel") ' Creates a comment in the first (raw) channel.

'Add values to Data Pad

Call Doc.AddToDataPad ()

'Search in previous direction for ECG marker again

ChannelIndex = 0

SetAction = kSetActivePoint

SelectMode = kSelectAround

SelectTime = 1

DataDisplayMode = kViewDataVisible

SelectAll = True

Direction = kSearchBack

FindType = "Search for event marker"

FindData = "JustThisChannel=1;"

Call Doc.Find (ChannelIndex, SetAction, SelectMode, SelectTime, DataDisplayMode, SelectAll, Direction, FindType, FindData)

'Search forwards for Finger first derivative

ChannelIndex = 7

SetAction = kSetToPreviousPoint

SelectMode = kSelectAround

SelectTime = 1

DataDisplayMode = kViewDataVisible

SelectAll = True

Direction = kSearchForward

FindType = "Search for event marker"

FindData = "JustThisChannel=1;"

Call Doc.Find (ChannelIndex, SetAction, SelectMode, SelectTime, DataDisplayMode, SelectAll, Direction, FindType, FindData)

'Add values to Data Pad

Call Doc.AddToDataPad ()

'Search in previous direction for ECG marker again

ChannelIndex = 0

SetAction = kSetActivePoint

SelectMode = kSelectAround

SelectTime = 1

DataDisplayMode = kViewDataVisible

SelectAll = True

Direction = kSearchBack

FindType = "Search for event marker"

FindData = "JustThisChannel=1;"

Call Doc.Find (ChannelIndex, SetAction, SelectMode, SelectTime, DataDisplayMode, SelectAll, Direction, FindType, FindData)

'Search forwards for Finger second derivative

ChannelIndex = 8

SetAction = kSetToPreviousPoint

SelectMode = kSelectAround

SelectTime = 1

DataDisplayMode = kViewDataVisible

SelectAll = True

Direction = kSearchForward

FindType = "Search for event marker"

FindData = "JustThisChannel=1;"

Call Doc.Find (ChannelIndex, SetAction, SelectMode, SelectTime, DataDisplayMode, SelectAll, Direction, FindType, FindData)

'Add values to Data Pad

Call Doc.AddToDataPad ()

Next

‘End

# DATA PROCESSINGTTINGS, AND MACROS

Data was sampled at 1000 Hz using the Powerlab data acquisition device (30 series, ADInstruments, CO) and was recorded, processed, and analyzed using LabChart 8.1.13 (ADInstruments) software. The ECG did not require any post-processing, but low-pass (50 Hz cut-off frequency) and mains filters on the ECG signal were used to ensure distinct R-wave peaks. When appropriate, detection thresholds inherent to LabChart (e.g. number of standard deviations from mean) were adjusted to ensure correct detection of the PPG signal. The data collection settings for LabChart files included i) four channels to collect each of the raw data signals for ECG and PPG, ii) 1^st^ and 2^nd^ derivative channels corresponding to each raw PPG signal channel, and iii) cyclic measurement channels corresponding to each raw, 1^st^ and 2^nd^ derivative channel which enabled detection of peaks for offline PAT determination using a custom macro. The macro computed PATs across three consecutive cardiac cycles, which is similar to the in-built Vicorder algorithm. In order to coordinate PPG and Vicorder PWV measurements (including for both Base and Tilt conditions), a marker was placed in LabChart at the moment when the Vicorder measurement was initiated. The PAT-deriving macro was initiated at these markers so that the corresponding LabChart PATs obtained from the three cardiac cycles were time-aligned precisely with the Vicorder measurements. The average of the closest two PATs were used for the main analyses.

Data quality was ensured by visually inspecting the peaks identified (R wave and 2^nd^ derivative) and transit times computed by the macro. If the data quality was not sufficient (e.g. poor ECG or PPG signal prevented ability to discern true peak), the next closest, subsequent cardiac cycles were utilized. However, if poor signal quality prevented the ability to align PPG and Vicorder measurements within one minute (e.g. if accurate PATs could only be obtained 70 seconds following the Vicorder measurements), or if the experimental protocol necessitated a time-sensitive positional change that precluded waiting one minute, then that particular PAT (and corresponding PWV) was omitted. Additional methodological details pertaining to data reduction and analysis including specific channel settings (e.g. sampling frequency, data filters) and data reduction processes are described in **Table S1**. The macro code is shown above.

### **MEASUREMENT CONSIDERATIONS**

For our PPG measures, PAT was calculated using a custom macro as the time from the R wave peak to the 2^nd^ derivative peak of the raw waveform. The R wave peak was used as the starting point of the PAT as it more closely aligns with ventricular ejection and the beginning of the pressure wave than the initial upstroke of the R wave, which signals the beginning of electrical and not mechanical left ventricular action [26]. This differs from the TT time component used in the referent PWV calculation, which does not include the pre-ejection period. As expected, the intrinsic inclusion of the pre-ejection period in our PPG measure of PAT (e.g. originates from ECG rather than pressure waveform), as well as slightly different distal sites of the PWV path lengths (e.g. ankle vs. toe and wrist vs. finger), contributed to the lack of absolute agreement between PPG and referent devices (cwPWV vs. hfPWV: $\sqrt{\mathrm{MSD}}=3.62$; caPWV vs. htPWV: $\sqrt{\mathrm{MSD}}=3.33$). While the purpose of this study was not to determine clinical relevance of PPG measures, we do acknowledge that the inclusion of PAT in PPG-derived PWV (unlike traditional tonometric and oscillometric methods) must be evaluated in future inquiries aimed at examining clinical implications of PPG-derived PWV.

To calculate PAT, the time-component of the PPG PWV calculation, we used the time between the R-wave and the 2^nd^ derivative peak of the raw PPG waveform as the 2^nd^ derivative peak closely aligned with the true upstroke (**Figures 1** and **S2**). The 2^nd^ derivative of the distal pressure wave upstroke has been used previously in traditional tonometric (e.g. SphygmoCor, Atcor Medical) and oscillometric devices for calculating TT including the Vicorder [24,25,41]. The utility of the 2^nd^ derivative in waveform analyses to identify the foot (upstroke) of a waveform is also evident based on its incorporation into calculations for several PPG-based stiffness indices (e.g. dicrotic elasticity index, aging index) related to PAT and pressure wave hemodynamics [42–44]. Other strategies besides use of the 2^nd^ derivative for calculating PAT were also considered. For example, the raw signal and 1^st^ derivative were trialed which produced more pronounced waveforms, yet did not closely align with the true peak and ultimately provided inferior agreement with the referent device (**Table S2**). Similarly, 3^rd^ and 4^th^ derivatives were trialed but were not usable due to excess nois
